# On the Treatment of Fracture of the Neck of the Thigh Bone, within the Capsular Ligament

**Published:** 1842-07-30

**Authors:** 


### ANALECTA.

*On the Treatment of Fracture of the Neck of the Thigh-Bone, within the Capsular Ligament.* By a Medical Officer of the Army.—I believe it will be generally admitted that no subject of surgical treatment has been more unsuccessful in its results than in cases of fracture of the neck of the femur; and it has appeared to me, that the explanations or apologies for our failures in such cases has been any thing but satisfactory, or even in accordance with what is known and regularly inculcated, of the effect of pressure on the most solid parts of the animal frame. At present I merely allude to those cases of failure which have been imputed to the circumstance of the fracture having been within the capsular ligament, and that there, that membrane intervening between the fractured portion of the bone, prevents the ossification of the fractured parts. I shall not notice what is generally known and assented to, that pressure against such an intermediate substance would most certainly, in a person of a sound constitution, occasion in time the absorption of the intervening obstacle to the re-union of the bone; but I will venture to affirm, that even cases of fracture of the neck of the femur within the capsular ligament, although the person injured would become immediately lame, still the distortion of the limb, at the seat of the injury, would not be easily ascertained even by the most practised and discerning examination, unless the fracture had been occasioned by extraordinary external violence.

In cases of the description which I now speak of, practitioners seem to me to have lost sight, and never considered the import and utility, of the ligamentum rotundum which binds the head of the femur to the acetabulum. Without this wonderful piece of mechanism, a person moving in the horizontal position, or even in walking upon an uneven surface, would be in continual danger of dislocating the femur. In this view of the matter, I therefore maintain, that, as the humble servants of nature, we should endeavor to follow her dictates in presuming to rectify any irregularity that happens in the wise and beneficent purposes of Providence. It is probably, in a great measure, owing to the rupture of the round ligament in cases of dislocation of the thigh, that a certain lameness always inconveniences the sufferer after it has been reduced, attended with a considerable swelling or fulness of the muscles adjoining the trochanter, particularly on every attempt to move the limb. If the opinion thus surmised be correct, it will be easily understood that, on a fracture of the neck of the femur taking place, the body of the bone being thereby disconnected with the acetabulum, it will, by its weight, naturally recede from the broken portion within the acetabulum, even by the best contrived bandaging for securing the apposition of the fractured parts, unless some means are had recourse to in order to support and maintain the trochanter in its healthy position: it is from neglect to this part of the treatment, as commonly practised, that I am inclined to suppose that in a great degree our failure in obtaining a cure in such cases of fracture is to be imputed; for I think that in a just apposition of the fractured ends of the bone no intervening substance could possibly prevent an ultimate re-union of the parts. I shall now, in a summary way, state a case or two in proof of the propriety of the practice I have hinted at: the parties being my friends, although not under my care, every circumstance of their cases was very well known to me.

The first is the wife of a physician, a lady past the meridian of life, weakly, of a spare habit: had her right thigh-bone broken at the neck by something taking her foot as she walked through the drawing-room: when laid upon the sofa, her husband could not perceive any particular injury where she complained of pain; and in this state she was carried to bed: she passed the night in much pain in the part; and it was not till from twenty to thirty hours elapsed that the nature of the accident was ascertained. The fracture was reduced when she had been laid on a firm mattrass; a firm bandage was tightly bound round the pelvis, and the limb being stretched in a straight line was bound in such a manner as to prevent all motion in it. She luckily escaped any feverish symptom; but in the course of a few weeks her husband got a cushion made to slip under the back, in order to relieve the intolerable pain and uneasiness she experienced from laying so long in the recumbent posture upon an unyielding surface: this cushion also gave support to the trochanter, and happily, at the end of four months, on the bandages being taken off, a perfect re-union of the bone was found to have taken place. This lady is now perfectly well, and the lameness but very little to be noticed.

The next case I shall mention is the wife of a colonel in our army. She is about the same age as the above, of a full habit, subject to inflammatory affections of the chest, and has suffered from gout: the neck of the thigh-bone was fractured, by her being blown over in a high wind when walking on an uneven surface. Her accident happened a month or two previously to that of the case above mentioned: they were attended by the same practitioner, and the same mode of treatment adopted—the bandaging of the outstretched limb, &c. &c. This lady had occasion to be bled and leeched during the first months of her confinement; but unluckily, on the bandages being removed at the end of four months, it was found that the bone had not only not united, but that the fracture was in every way as upon the occurrence of the accident. Upon this occasion her hasband was earnestly recommended to have a similar cushion used as in the former case, upon the fracture being reduced; and to the satisfaction of all her friends it was found, on removing the bandages, after laying four months more in the recumbent posture, that the bone was perfectly united: perhaps an unique case in the annals of surgery, that a bone should unite after several months' separation. I have seen this lady repeatedly since her cure: the motion of the hip-joint is perfect; but although the knee-joint is not affected, in consequence of the straight position in which the limb was placed, and the bandaging, the muscles have thereby lost their natural action; and from this cause she cannot bend her leg. The heel is also drawn up, owing, as I suppose, to so long confinement in one posture, and the consequent contraction, and perhaps the conglutination, of the fibres of the flexor muscles. In other respects she enjoys her usual health.

I shall next mention the case of a lady about forty years of age, the wife of a surgeon to an hospital. The accident occurred when playing at shuttle-cock with her children in the drawing-room. In this case, also, the nature of the injury was not immediately discovered. When I saw her, a few days after, she was lying in a soft bed, with a bandage round the pelvis; the limb laid straight, but not bandaged.

In this case, I have no doubt, the trochanter was supported and maintained in its natural position by the pressure of the mattrasses of the bed, which were not firm, as in the former cases, but soft and yielding: this lady very soon recovered the use of her limb, without any remarkable lameness. I could, to the above, add other cases, where, I believe, a fracture of the neck of the thigh-bone had taken place within the capsular ligament, and the protuberance of the fractured bone not being discernible on the first examination, the disease has been treated as a severe bruise, and which terminated in permanent misery and lameness; or if the true nature of the injury has at length been discovered, the failure of the cure has been imputed to the intervention of the capsular ligament between the ends of the broken bone. The inference I draw from the foregoing statement is, in the first place, that every means should be adopted for securing the apposition of the fractured ends of the bone, and that this view is mainly to be effected by supporting the great body of the femur, which otherwise, from its weight, must recede from and sink under the surface of the broken portion, remaining fixed in the acetabulum; an inspection of the skeleton must satisfy the most superficial that such will be the consequence when the person, having the neck of the femur broken, is laid upon his back. I do not see the necessity of placing the patient, in this state, upon a hard, unyielding mattrass. I know it often occasions intolerable distress to the patient, and may therefore protract, if not interrupt, the cure: a mattrass in ordinary use will obviate many of these inconveniences. The fracture being reduced, the limb should be laid in a bent position, as in other fractures of the femur, so that all the muscles may be in a state of complete relaxation; and after the bandage is firmly secured round the pelvis, a long cushion of horse-hair, about six or eight inches in breadth, according to the bulk of the patient, and about the same thickness, and so long as to reach from the under and upper part of the os ilium, and going under the trochanter to a few inches below it. This cushion should be secured with tapes at each end to fix round the body and the thigh, and should be carefully examined every day to see that it does not shift its place: or to avoid the chance of this happening, the bandage around the pelvis might be so constructed as to give the necessary support to the thigh. In this way the cure of such cases will be more certain, and require less time than the practice now in general use.—*London Med. Gaz.*, May 20, 1842.

